# Hematological Manifestations of SLE at Initial Presentation: Is It Underestimated?

**DOI:** 10.5402/2012/961872

**Published:** 2012-07-09

**Authors:** P. K. Sasidharan, M. Bindya, K. G. Sajeeth Kumar

**Affiliations:** Department of Medicine, Calicut Medical College, Kozhikode, Kerala 673008, India

## Abstract

SLE can present with hematological manifestations alone or along with features of other system involvement. With a low index of clinical suspicion or inadequate follow up the diagnosis may be delayed or missed at the time of presentation, in those with hematological abnormalities as the initial manifestation. An observational study was conducted among patients of SLE, in a tertiary referral centre of North Kerala, with the purpose of estimating the proportion of patients with hematological manifestations as the initial presentation of the disease and to study their nature. It was observed that 82% of the patients had hematological manifestations at presentation. It is the most common presenting manifestation of SLE in people of North Kerala. Autoimmune hypothyroidism was one of the common coexisting abnormalities in these patients, which is not included in the American College of Rheumatology (ACR) criteria for diagnosis. Arthritis was uncommon among those who presented with hematological manifestations. A significant number of patients do not satisfy the ACR criteria at the time of diagnosis but do so on follow up. The ACR criteria are weak to diagnose such patients and therefore need revision. We therefore propose an alternative to ACR criteria as “Kozhikode criteria for SLE”.

## 1. Introduction 

Systemic Lupus Erythematosus (SLE) at its onset may involve one or more organ systems and over a time additional manifestations may appear after a variable period. The systems involved in SLE are musculoskeletal, cutaneous, renal, nervous system, hematological, vascular, pulmonary, gastrointestinal, and ocular. Hematological manifestations (abnormalities of the formed elements of the blood, of the clotting and fibrinolytic factors and related systems) of SLE are diverse and often they are the presenting manifestations of the disease [1–3]. The major hematologic manifestations of SLE are anemia, leukopenia, thrombocytopenia, and the antiphospholipid antibody syndrome (APLAS).

The hematological changes, though very commonly seen, are not properly evaluated or estimated and are not given enough representation in the American College of Rheumatology (ACR) criteria for diagnosis of SLE. It is only natural to expect hematological manifestations more often than others, since blood and blood vessels together contain more diverse number of antigens than any other organ in the body and in SLE auto antibodies are known to develop against any antigen or tissue. It has been our observation since the last two decades that many cases of SLE present with hematological abnormalities alone, without features of musculoskeletal, skin, or other system involvement [1]. In some of these cases presenting with anemia, thrombocytopenia, pancytopenia, or thrombotic episodes, especially so in young females, the diagnosis may be delayed or initially missed if the index of suspicion is low or if there is improper and inadequate followup [1]. Many cases which present initially with manifestations due to involvement of any one tissue or organ alone (autoimmune hemolytic anemia, lupus nephritis etc.) and some cases which are ANA negative do not satisfy the ACR criteria initially but do so on followup. In most of these cases empirical treatment could be started, to the advantage of the patient, if there is any evidence of an autoimmune phenomena, after ruling out other differential diagnoses. In such cases response to treatment or the development of other features of the disease on followup confirms the diagnosis of SLE. 

This study was conducted to estimate the proportion of patients with hematological abnormalities as the initial manifestation of SLE and to study the nature of these various hematological problems. 

## 2. Materials and Methods

Hematological manifestations at presentation in patients with SLE were evaluated by an observational study design. All newly diagnosed SLE cases and previously diagnosed cases under followup, during the study period of 12 months (April 2009–March 2010) in the departments of General Medicine, Hematology, Rheumatology, Nephrology and Dermatology of Government Medical College Kozhikode (which is the chief referral centre of North Kerala) were included in the study. Data was collected by a structured personal interview and detailed clinical examination. Basic investigations consisting of complete blood counts including red cell indices and ESR, renal and liver function tests, urine routine, peripheral smear, ANA, and Anti-dsDNA were done for all cases. Further relevant investigations like reticulocyte count, Coomb's test, ANA profile, radiological tests, tissue biopsy, or cytology including bone marrow examination were done in individual cases as and when indicated.

 All patients included in the study satisfied either the American College of Rheumatology criteria for the definition of SLE or the new criteria evolved by us (based on our own observations of SLE over last two decades) and were utilized for the purpose of the study (Kozhikode Criteria). The new criteria was essential to include some of those patients who did not initially satisfy the ACR criteria at the time of inclusion but did so on followup.

### 2.1. The Kozhikode Criteria for Diagnosis of SLE [1]

#### 2.1.1. Two Essential/Major Criteria

1. Presence of an unresolved autoimmune disorder which is known to occur with SLE (e.g., Chronic ITP, autoimmune hemolytic anemia, skin lesion, Antiphospholipid antibody syndrome (APLAS), autoimmune hypothyroidism, autoimmune hepatitis).
2. No other cause other than autoimmunity for the said clinical problem: by clinical reasoning and investigations.

#### 2.1.2. Minor Criteria

1. Another coexisting autoimmune disorder/any other additional evidence of autoimmunity.
2. Positive ANA.
3. Positive Anti-ds DNA.
4. Sustained and definite response to treatment with steroids and immunosuppressant even after six months of followup.

The diagnosis of SLE was made if the patients satisfied the presence of two essential criteria along with any two of the four minor criteria given above. We have been using these criteria for the last two decades, and the followup had proved that they were all SLE and subsequently satisfied the ACR criteria as well. But it was not validated by any organized study protocol. 

Data entry was done by using Epi info software and Microsoft excel. Data was analyzed by standard statistical techniques with SPSS.

## 3. Results

One hundred and eight patients satisfied the inclusion criteria and were included in the study. Out of which 53 patients were newly diagnosed and the rest 55 were previously diagnosed cases under followup during the study period. Male : female ratio in the whole of the study subjects was 1 : 10. The mean age of females was 30 (SD-10) and that of males was 34.5 (SD-18.5). Majority of the females were in the age group of 16–35 years, while the male subjects were almost evenly distributed in the various age groups. Five patients (4.6%) were aged more than 50 years. The male : female ratio in this age group was 4 : 1.

**Figure 1:**
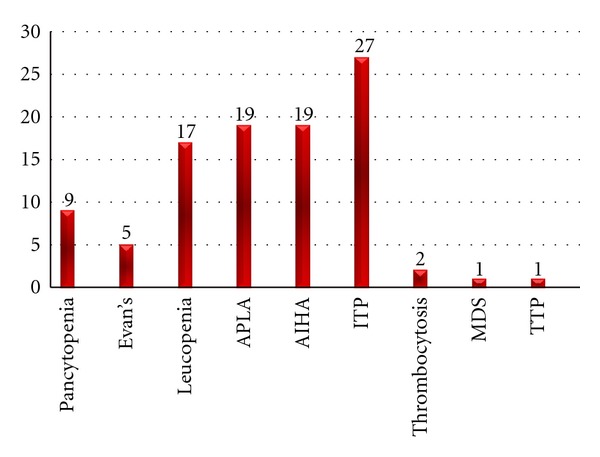
The hematological manifestations at presentation.

 Eighty nine patients (82%) had hematological manifestations at presentation out of which thirty-eight had only hematological abnormality as the first manifestation. The next common presentation was arthritis in 44 cases (40.7%) followed by lupus nephritis in 25 cases (23 %) (Table 1 and Figure 1). 

 Among those subjects presenting with hematological abnormality, coexisting renal involvement was present in 19 patients and musculoskeletal involvement in the form of arthritis or arthralgia was seen in 12 only. On statistical analysis, a significant inverse association was found between the presence of musculoskeletal and hematological manifestations (*P* value <0.001). Autoimmune hypothyroidism (*n* = 10) and autoimmune hepatitis (*n* = 6) were the next common associated manifestations in patients with initial hematological presentation, but they are not included in the ACR criteria for SLE.

 The most prevalent hematological abnormality was anemia, present in 62.9% of the patients in the study group with a mean hemoglobin value of 9.5 mg/dL. (hemoglobin level <12.5 mg/dL in females and <11.5 mg/dL in males was taken an anemia). Morphologically normocytic normochromic anemia was the most common type (53%). But anemia was multifactorial like autoimmune haemolysis, iron deficiency, folic acid deficiency, anemia of chronic inflammation, etc.). Autoimmune hemolytic anemia was seen in 27.9% of them. Thrombocytopenia was present in 39.8% and leukopenia in 15.7%. 

**Figure 2:**
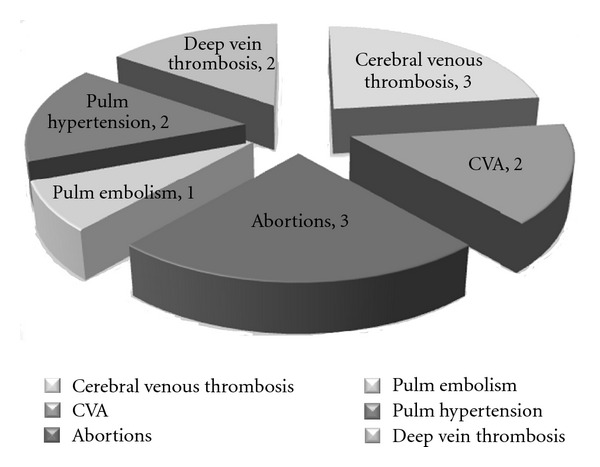
Presentation of antiphospholipid antibody syndrome.

 The most common hematologic diagnosis at presentation was immune thrombocytopenia (27 cases) followed by autoimmune hemolytic anemia and antiphospholipid antibody syndrome (19 cases each) (Figure 1). Cerebral venous thrombosis and recurrent abortions were the most common presenting feature of antiphospholipid antibody syndrome (Figure 2). Coexisting thrombocytopenia was present in 7 out of the total 19 patients (36.8%) with APLA syndrome and one patient had associated autoimmune hemolytic anemia. Thrombocytopenia in APLAS was mild and benign and was not associated with bleeding; these patients did not require treatment for thrombocytopenia. There were two cases (1.8%) with thrombocytosis at presentation, one of which was a case of APLAS.

 Anti nuclear antibody test was negative or ACR criteria was not satisfied at the time of presentation in 12 patients (11%) (Table 2). The maximum latency to ANA positivity or satisfaction of ACR criteria was 12 years, in a patient of APLA syndrome with cerebral infarcts who had an initial presentation as isolated thrombocytopenia 12 years back and was ANA negative at that time. 

## 4. Discussion

In this study, hematological manifestations were found to be the most common initial presentation of SLE and it was the presenting manifestation in 82% of the subjects. This observation was made in 108 patients both newly diagnosed patients and those undergoing treatment during the study period of one year in a tertiary care centre in North Kerala. This observation is contradictory to the description of the disease in most Western and Indian text books [4, 5] and majority of the previously conducted studies [6, 7]. However, a multicentre French study and a Turkish study on the initial presentation of childhood onset lupus showed that the most common initial manifestation was hematological [8, 9]. This finding supports our observation and emphasizes the fact that hematological manifestations are a common presentation that may be missed if the index of suspicion is low.

Several studies conducted in different parts of the world have evaluated the hematological findings in SLE and the prevalence rates obtained in them are almost comparable with that of ours with anemia being the most common hematological manifestation [9–12]. But hematological manifestation as the initial presentation and its proper inclusion in the criteria for diagnosing SLE were not addressed in any of the studies.

 A coexisting musculoskeletal manifestation was present in only 12 of the subjects who presented with a primary hematological problem, and a significant inverse association was found between the presence of musculoskeletal and hematological manifestations. The commonly coexisting abnormality of autoimmune hypothyroidism in patients presenting with hematological manifestations is also not represented in the ACR criteria. In a study done in Netherlands published in 1991, serositis was found to be less common with hemolytic anemias [9] while no studies comparing the coexistence of arthritis and hematological manifestations were found in literature.

Coexisting thrombocytopenia was present in 7 out of the total 19 patients (36.8%) with APLA syndrome. In a study conducted by Caudrado et al approximately 25% of patients with antiphospholipid antibody syndrome (APLAS) was found to have thrombocytopenia. 

Twelve patients (11%) were tested negative for ANA and did not satisfy the ACR criteria at the time of presentation but did so on followup. Thus ANA negativity does not rule out SLE in its early stages. They all had the clinical diagnosis of possible SLE or evolving SLE at the time of initial presentation itself. In 1982 McHardy et al. investigating a cohort of SLE patients in Aberdeenshire, had suggested a prevalence of 8.9% for ANA-negative SLE [13]. Gladman et al. and Ferreiro et al., in two separate studies found a prevalence of approximately 5% of SLE cases which were ANA negative at the time of diagnosis [14, 15]. This emphasizes the importance of relying on individualised clinical judgement rather than on existing criteria alone for treating the disease and the need for rigorous followup of all suspected cases even if they are ANA negative initially. Needless to say the criteria for diagnosis of SLE need revision to make an early diagnosis in such patients for which we are proposing the new criteria (Kozhikode criteria) as utilized in this study.

## 5. Conclusions

Hematological manifestation is the most common presenting manifestation of SLE in people of North Kerala, India. Thrombocytopenia, hemolytic anemia, and antiphospholipid antibody syndrome (APLAS) were the common presentations. The most common hematological abnormality over the entire course of the disease was anemia which was multifactorial. There was no significant association of arthritis with hematological manifestations. Autoimmune hypothyroidism, which is not included in the ACR criteria, is a common coexisting abnormality in patients with initial hematological manifestation. A significant number of patients do not satisfy the ACR criteria at the time of diagnosis but do so on followup. We are proposing a new criteria for early diagnosis of SLE which we would call as “Kozhikode criteria” to include the observations made by us.

## Figures and Tables

**Table 1:** Clinical features at the initial presentation of SLE.

Manifestations	Number	Percentage
Hematological		
Anemia	68	63
Thrombocytopenia	43	40
APLAS	19	17.5
Leukopenia	17	16
Others		
Arthritis	44	31
Renal (lupus nephritis-20/renal tubular acidosis-5)	25	23
Dermatological	20	18.5
Fever	9	8
Neurological	8	4
Ocular (episcleritis)	3	3
Pulmonary	3	3
Endocrine	15	14
Hypothyroidism (13) Addison (1) Graves (1)		

**Table 2:** Cases which were initially ANA negative, with the time period to subsequent positivity on prospective and retrospective followup.

Case no.	Diagnosis	App. time to ANA positivity or satisfying ACR criteria
(1)	Immune thrombocytopenia (ITP)	32 months
(2)	Immune thrombocytopenia	22 months
(3)	Immune thrombocytopenia, hypothyroidism	40 months
(4)	Autoimmune hemolytic anemia	7 months
(5)	Autoimmune hemolytic anemia, hepatosplenomegaly, nephrotic syndrome	11 months
(6)	ITP, APLA, severe PAH, high ESR	144 months
(7)	APLA, high ESR	8 months
(8)	Anemia, splenomegaly, Sjogren	6 months
(9)	Secondary Sjogren	4 months
(10)	Thyrotoxicosis, (ITP)	11 months
(11)	Polyarthralgia	8 months
(12)	Polyarthralgia, elevated ESR, anemia	4 months
